# Resilience in Pediatric-Onset Inflammatory Bowel Disease: Associations with Age, Therapy Change, and Health-Related Quality of Life

**DOI:** 10.3390/children12081062

**Published:** 2025-08-13

**Authors:** Elizabeth Hilow, Jessica Barry, Nila Mistry Ambani, Kate Eshleman, Sarah Worley, Wei Liu, Jacob A. Kurowski

**Affiliations:** 1 Division of Pediatric Gastroenterology, Hepatology and Nutrition, Cleveland Clinic Children’s, Cleveland, OH 44195, USA; 2 Center for Pediatric Behavioral Health, Cleveland Clinic Children’s, Cleveland, OH 44195, USA; 3 Department of Quantitative Health Sciences, Cleveland Clinic, Cleveland, OH 44195, USA

**Keywords:** inflammatory bowel disease, resilience, quality of life

## Abstract

**Highlights:**

**What are the main findings?**

- Resilience in pediatric IBD is impacted by changes in therapy and varies with age.
- Resilience in pediatric IBD is independent of disease duration and quality of life.

**What is the implication of the main finding?**

- Assessment of resilience may identify at-risk patients and guide strategies to improve coping skills.

**Abstract:**

Background: Resilience is associated with improved outcomes in adult inflammatory bowel disease (IBD), yet little is known about its relationship to health-related quality of life and disease characteristics in pediatric-onset IBD. Methods: This prospective, cross-sectional study enrolled pediatric-onset IBD patients (≥12 years) at Cleveland Clinic Children’s. Participants completed the Connor–Davidson Resilience Scale (CD-RISC-10) and an age-appropriate health-related quality of life (HRQOL) survey. Measure: IMPACT-III (ages 12–17) or SF-36 (≥18). Demographic and clinical data were collected via chart review. Associations between resilience, HRQOL, and clinical variables were analyzed. Results: Seventy participants completed the study (35 adolescents and 35 young adults). Young adults had significantly higher resilience scores than adolescents (31 ± 4.6 vs. 27 ± 5.3; *p* = 0.007). Resilience scores were significantly lower among patients who had experienced a change in IBD therapy within the prior year (27 vs. 30; *p* = 0.045). No significant associations were found between resilience and age at diagnosis, disease duration, HRQOL, or prior surgery. Use of pharmacologic treatment for mental health conditions was higher in young adults compared to adolescents (22.9% vs. 14.3%; *p* = 0.015), despite similar rates of diagnosed mental health comorbidities. Conclusions: Resilience in pediatric-onset IBD patients varies by age and is lower in the context of recent therapy changes, suggesting a potential vulnerability during periods of disease instability. Routine assessment of resilience may help identify patients who could benefit from early psychosocial intervention to support coping and improve long-term outcomes.

## 1. Introduction

Inflammatory bowel disease (IBD) is a chronic condition affecting over 2 million people in the United States, with rising incidence levels in children. Approximately 25% of patients with IBD are diagnosed before age 20, and pediatric-onset disease is associated with prolonged disease burden and greater cumulative exposure to medical and psychological challenges [1,2].

Resilience is defined as an individual’s adaptive capacity to cope with and recover from adversity. In adults with IBD, higher resilience has been associated with improved health outcomes, including lower disease activity, increased quality of life, and fewer surgeries [3]. Emerging evidence also suggests that resilience may play an important role for adolescents with IBD, particularly during the transition from pediatric to adult care [4]. The Connor–Davidson Resilience Index (CD-RISC) is a validated instrument for measuring resilience and has been applied in both pediatric (≥12 years) and adult patients with IBD and other chronic diseases [5].

Health-related quality of life (HRQOL) specifically measures the impact of chronic disease on physical, psychologic, and social health, offering a comprehensive view of patient well-being [6]. It reflects the patient’s perception of how chronic disease impacts daily life and is an important outcome measure in pediatric IBD care. While positive psychology interventions have been proposed to enhance patient resilience and improve HRQOL, the relationship between these constructs in pediatric IBD remains poorly defined.

The primary aim of this study was to identify demographic and health-related factors influencing resilience in pediatric IBD patients and examine its relationship with HRQOL. We hypothesized that lower resilience would be associated with clinical instability, mental health comorbidities, and lower HRQOL scores.

## 2. Methods

### 2.1. Research Design

This prospective, cross-sectional study included patients with pediatric-onset IBD treated at Cleveland Clinic Children’s (CCC) and was approved by the Cleveland Clinic Institutional Review Board in accordance with the Declaration of Helskini. Participants were recruited based on the following inclusion criteria: (1) established care in Pediatric Gastroenterology at CCC; (2) confirmed diagnosis of IBD for at least 6 months; (3) age ≥ 12 years; and (4) ability to read and comprehend English.

### 2.2. Procedures

Informed consent was obtained from all subjects involved in the study. Participants received a unique QR code linking to a secure REDCap survey, accessible on their personal device. The survey included the CD-RISC-10 and an age-appropriate HRQOL survey. Alternatively, participants could receive the survey link through the hospital’s electronic medical record patient portal. For those that did not complete the surveys at the time of consent, the QR code was reissued during a subsequent clinic visit.

A chart review was conducted for all participants who completed the study surveys to collect demographic and health-related data. Health-related variables included disease duration and phenotype, treatment, surgical history, steroid or exclusive enteral nutrition (EEN) use in the prior year, IBD-related imaging, endoscopic findings (if performed within the prior year), laboratory values within 3 months of the visit, healthcare utilization, and documented mental health comorbidities (anxiety, depression, or ADD/ADHD). Healthcare utilization was identified by the need for a visit to the emergency department (ED) or hospitalization within the past year.

Mental health treatment, defined as pharmacologic therapy or engagement with mental health providers, was also recorded. For participants who underwent a colonoscopy in the previous year, images were independently reviewed by the study team using the validated scoring systems the Mayo endoscopic sub-score for ulcerative colitis and the Simplified Endoscopic Mucosal Assessment for Crohn’s Disease (SEMA-CD) [7,8].

### 2.3. Measurements

Three validated instruments were used to assess resilience and HRQOL. Resilience was measured using the 10-item Connor–Davidson Resilience Index (CD-RISC-10), scored on a 5-point Likert scale with higher scores indicating greater resilience [5]. HRQOL was assessed with age-appropriate questionnaires. Adolescents (ages 12–17 years) completed the IMPACT-III, a 35-item IBD-specific tool scored on a 5 pt Likert scale [9]. It evaluates HRQOL over the preceding two weeks across four domains: general well-being, emotional functioning, social functioning, and body image. Young adults (≥18 years) completed the Short-Form Health Survey (SF-36), a widely used 36-item questionnaire scored on a 2 to 6 pt Likert scale [10,11,12]. It assesses a time period of two weeks prior to survey completion across eight domains: physical functioning, bodily pain, role limitations due to physical health or personal/emotional problems, emotional well-being, social functioning, energy/fatigue, and general health perception. It is one of the most used HRQOL surveys in adult patients with IBD.

### 2.4. Statistical Methods

Data were described using medians and quartiles for continuous variables and counts and percentages for categorical variables. Analyses of HRQOL were performed separately for patients who completed the IMPACT-III and the SF-36. The associations between IMPACT-III, SF-36, and CD-RISC 10 scores were assessed using Spearman correlation coefficients with 95% confidence intervals. Univariable associations between demographic and clinical characteristics and CD-RISC 10 scores were assessed using Spearman correlations (continuous and ordinal characteristics) and Kruskal–Wallis tests (categorical characteristics). All tests were two-tailed and performed at a significance level of 0.05. SAS 9.4 software (SAS Institute, Cary, NC, USA) was used for all analyses.

## 3. Results

### 3.1. Demographics and Disease Status

A total of 91 eligible participants were identified and consented for the study, 70 completed the assigned surveys. The cohort included 35 adolescents (20 females and 15 males) and 35 young adults (14 females, 21 males). The majority of participants (83%) had CD (Table 1). Median age at diagnosis was significantly higher in young adults compared to adolescents (13 vs. 12 years; *p* = 0.029) and median disease duration was longer in young adults compared to adolescents (7.1 vs. 2.9 years; *p* < 0.001). There was no significant difference between groups in current medication use, steroid use in the past year, surgical history, or therapy changes within the past year. The majority of patients were currently receiving an infusion-based biologic, with no difference between adolescents (94%) and young adults (91%) (*p* = 0.99). No patients received EEN in the previous year. Markers of disease activity, including endoscopic scores (*p* = 0.40), inflammatory markers (*p* = 0.98), hemoglobin (*p* = 0.10), or albumin (*p* = 0.95) did not differ significantly between groups.

### 3.2. Resilience and HRQOL

There was no significant difference in the prevalence of documented mental health comorbidities (anxiety, depression, and ADD/ADHD) between adolescents and young adults (Table 1). However, use of pharmacotherapy for mental health conditions was significantly higher in young adults compared to adolescents (22.9% vs. 14.3%; *p* = 0.015).

**Figure 1:**
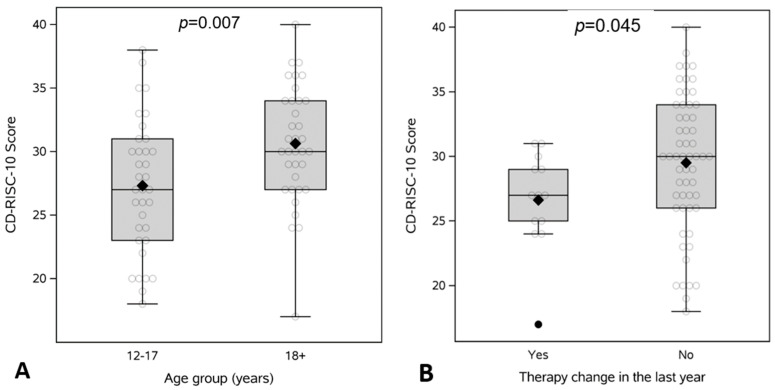
CD-RISC-10 Resilience scores comparing (**A**) adolescents (12–17 years) to young adults (≥18) and (**B**) presence of therapy change in the prior year.

Resilience scores were significantly higher in young adults compared to adolescents (31 ± 4.6 vs. 27 ± 5.3; *p* = 0.007) (Figure 1A). Across all participants, those who experienced a change in IBD therapy within the past year (n = 13) had significantly lower resilience scores compared to those with stable therapy (Δ in therapy = 27 vs. No Δ in therapy = 30; *p* = 0.045) (Figure 1B). There was no significant correlation between resilience and age at diagnosis (rho = 0.21; *p* = 0.07) or duration of disease (rho = 0.17; *p* = 0.16) (Supplementary Table S1). Resilience was also not associated with HRQOL or prior surgery (Supplemental Tables S1–S3).

## 4. Discussion

Patients with IBD, particularly youth, face a life-long disease burden with both physical and psychological challenges. Resilience is increasingly recognized as a protective factor. Sehgal et al. found an association between higher resilience and lower disease activity in adults with IBD [3]. In this study of pediatric-onset IBD patients, we found that resilience varied by age group, with young adults demonstrating significantly higher resilience scores than adolescents. This difference may reflect psychological development, intellectual maturity or increased adaptation over time. Importantly, resilience was not associated with age at diagnosis or disease duration, suggesting that individual factors may play a more central role in psychological adaptation. As such, fostering resilience may offer a valuable pathway to support psychological well-being and disease self-management over time.

A key finding was the association between lower resilience scores and recent changes in IBD therapy. Patients who required a therapy change within the prior year, likely reflecting increased disease activity or complications, had significantly lower resilience scores. This finding supports the hypothesis that resilience may be sensitive to disease instability or perceived health uncertainty. Although the directionality cannot be established in a cross-sectional design, it highlights resilience as a potential marker for vulnerability during periods of therapeutic transition.

Despite previous studies linking resilience to improved quality of life in adult IBD, we did not observe a significant correlation between resilience and HRQOL in our cohort [3]. This may reflect developmental differences in how resilience manifests or limitations in the sensitivity of current tools to capture subtle effects in the pediatric population. Additionally, though mental health comorbidities were similarly prevalent in both age groups, pharmacologic treatment was significantly more common in young adults, which may partially explain their higher resilience scores.

Measures of resilience can serve as valuable tools not only for psychologists but also for medical providers, offering a benchmark to build coping skills in our patients. In a prior study by Carlsen et al. identifying resilience as a predictor of transition readiness in adolescents and young adults with IBD, the mean CD-RISC-10 score was 30.4 in a cohort of 87 patients aged 16–23 years—comparable to the scores observed in our study [4]. Notably, both their findings and ours demonstrate mean resilience scores that fall below established general population norms (32.1 and 31.8, respectively) [13]. Given the elevated rates of anxiety and depression in this population, both of which are linked to worse disease outcomes and lower HRQOL, routine assessment of resilience may help identify patients at greater psychological risk [14]. By recognizing those with lower resilience and risk factors, providers can implement targeted strategies to strengthen adaptive capacity. Enhancing resilience may improve healthcare outcomes and facilitate critical milestones in care, including transition readiness [4].

This study has several limitations. First, its cross-sectional design limits the ability to establish causal relationships between resilience, HRQOL, and clinical variables. Second, the study was conducted at a single tertiary care center, which may limit generalizability to broader pediatric IBD populations, particularly those in community settings. Third, resilience and HRQOL were assessed using self-reported measures, which are subject to response and recall biases. Additionally, while validated tools were used, the IMPACT-III and SF-36 assess slightly different constructs, complicating direct comparisons between age groups. Finally, the modest sample size may limit the power to detect more nuanced associations between resilience and clinical outcomes. Future studies should consider the assessment of parental resilience, in addition to children’s, to assess the relationship between parental and child resilience.

Taken together, these findings suggest that resilience is a dynamic trait influenced by clinical status and possibly modifiable through targeted interventions. Early identification of low resilience, particularly during periods of therapy escalation, may offer a window for psychological support aimed at improving overall outcomes. Future longitudinal studies are needed to clarify causality and evaluate the impact of resilience-building interventions on both psychological well-being and disease trajectory in pediatric IBD.

## 5. Conclusions

This study highlights resilience in pediatric IBD as a meaningful, measurable trait that varies by age and is influenced by recent changes in therapy. While resilience was not directly associated with HRQOL or disease duration, its lower levels in adolescents and those undergoing therapy changes suggest an opportunity for early intervention. Routine assessment of resilience in clinical care may help identify at-risk patients and guide supportive strategies aimed at strengthening coping skills. Incorporating resilience-building interventions into multidisciplinary IBD care may ultimately improve long-term outcomes and support transition to adult IBD care.

## Figures and Tables

**Table 1:** Demographic and clinical characteristics by age group.

Factor	Adolescents 12–17 Years (*N* = 35)	Young Adults ≥18 Years (*N* = 35)	*p*-Value
	Statistics	Statistics	
Demographics			
Age	15 (12, 17)	20 (18, 26)	<0.001 ^b^
Female	20 (57)	14 (40)	0.15 ^c^
White	26 (74)	30 (86)	0.72 ^d^
IBD Medical History			
Age at diagnosis (yrs)	12 (4.0, 16)	13 (7.0, 19)	0.029 ^b^
Years since diagnosis	2.9 (0.59, 8.4)	7.1 (1.1, 16)	<0.001 ^b^
Crohn’s Disease	30 (86)	30 (86)	0.65 ^d^
Previous surgery related to IBD	8 (23)	9 (26)	0.78 ^c^
Current IBD-Related Medications: Infusion-based therapy	33 (94)	32 (91)	0.99 ^d^
Therapy change in the past year	6 (17)	7 (20)	0.76 ^c^
Steroid use in the past year for IBD	6 (17)	8 (23)	0.55 ^c^
ED visit for IBD in the past year?	2 (5.7)	2 (5.7)	0.99 ^d^
Hospitalizations in the past year for IBD	4 (11)	1 (2.9)	0.36 ^d^
Endoscopy in the past year?	13 (37)	15 (43)	0.63 ^c^
Mental Health			
Depression	1 (2.9)	3 (8.6)	0.61 ^d^
Anxiety	10 (29)	6 (17)	0.25 ^c^
ADHD/ADD	1 (2.9)	4 (11)	0.36 ^d^
None	22 (63)	27 (77)	0.19 ^c^
Other	1 (2.9)	0 (0)	0.99 ^d^
Pharmacotherapy for mental health diagnosis	5 (14)	8 (23)	0.015 ^d^
Mental health provider engaged in the last 6 months	8 (23)	4 (11)	0.20 ^c^
Survey Results			
CD-RISC-10 Score	27 ± 5.3	31 ± 4.6	0.007 ^a^
IMPACT-III Total Score	71 ± 12		
IMPACT-III Emotional Functioning Domain Score	63 ± 17		
IMPACT-III Social Functioning Domain Score	78 ± 14		
SF-36 General health		56 ± 21	
SF-36 Emotional well-being		69 ± 14	
SF-36 Social functioning		79 ± 18	

Statistics presented as Median (min, max), N (column %). *p*-values: ^a^ = Satterthwaite *t*-test, ^b^ = Wilcoxon Rank Sum test, ^c^ = Pearson’s chi-square test, ^d^ = Fisher’s Exact test.

## Data Availability

The raw data for this study were generated at Cleveland Clinic. Derived data supporting the findings of this study are available from the corresponding author upon request due to patient privacy.

## Supplementary Materials

The following supporting information can be downloaded at https://www.mdpi.com/article/10.3390/children12081062/s1, Supplementary Table S1: Correlation between clinical characteristics and CD-RISC-10 scores. Supplementary Table S2: Patients ages 12–17: Correlation between IMPACT III and CD-RISC 10 scores. Supplementary Table S3: Patients aged 18 and older: Correlation between SF-36 CD-RISC 10 scores.

## Abbreviations

Cleveland Clinic Children’s (CCC), Connor–Davidson Resilience Index (CD-RISC), health-related quality of life (HRQOL), inflammatory bowel disease (IBD), Short-Form Health Survey (SF-36).
