# Supplementary material for: Resilience in Pediatric-Onset Inflammatory Bowel Disease: Associations with Age, Therapy Change, and Health-Related Quality of Life
Source: Children (Basel). 2025 Aug 13;12(8):1062. doi: 10.3390/children12081062 (PMC12384607; doi:10.3390/children12081062)
Supplement: Supplementary file 1 [file children-12-01062-s001.zip › children-3780945-supplementary.pdf]

Supplemental Table S1. All patients: Correlation between clinical characteristics and CD-RISC-10 scores. Statistics presented as Median [25th, 75th percentiles] or Spearman's correlation (95% CI).

Factor	N	Statistics	p-value
**Age at diagnosis (years)**	70	0.21 (-0.02, 0.43)	0.074
**Years since diagnosis**	70	0.17 (-0.07, 0.39)	0.16
**Gender**			0.59
. **Male**	36	28.5 [26.0, 32.5]	
. **Female**	34	30.0 [26.0, 34.0]	
**IBD classification**			***0.022***
. **Crohn's Disease**	58	30.0 [26.0, 34.0]	
. **Ulcerative Colitis**	10	27.5 [24.0, 29.0]	
. **Indeterminate Colitis**	2	20.5 [18.0, 23.0]	
**Previous surgery related to IBD**			0.64
. **No**	53	29.0 [26.0, 32.0]	
. **Yes**	17	30.0 [26.0, 34.0]	
**Therapy change in the last year**			***0.045***
. **Yes**	13	27.0 [25.0, 29.0]	
. **No**	57	30.0 [26.0, 34.0]	
**Steroid use in the last year for IBD**			0.59
. **Yes**	14	29.0 [24.0, 31.0]	
. **No**	56	30.0 [26.0, 33.0]	
**Pharmacotherapy for mental health diagnosis?**			0.87
. **No**	57	32.0 [26.0, 35.0]	
. **Yes**	13	30.0 [26.0, 34.0]	
**Mental health provider engaged in the last 6 month**			0.43
. **No**	58	29.0 [26.0, 32.0]	
. **Yes**	12	30.0 [26.0, 34.5]	

Statistics presented as Median [25th, 75th percentiles] with Kruskal-Wallis test or Spearman's correlation (95% CI).

Supplemental Table S2. Patients ages 12-17: Correlation between IMPACT III and CD-RISC 10 scores.

Factor	N	rho (95% CI)	p-value
**IMPACT-III Body Image Domain Score**	35	0.26 (-0.08, 0.55)	0.13
**IMPACT-III Emotional Functioning Domain Score**	35	0.24 (-0.10, 0.53)	0.17
**IMPACT-III Social Functioning Domain Score**	35	0.17 (-0.17, 0.48)	0.33
**IMPACT-III Total Score**	35	0.22 (-0.12, 0.52)	0.20
**IMPACT-III Well-being Domain Score**	35	0.09 (-0.25, 0.41)	0.61

rho: Spearman's correlation; CI: confidence interval

Supplemental Table S3. Patients aged 18 and older: Correlation between SF-36 CD-RISC 10 scores.

Factor	N	rho (95% CI)	p-value
**SF-36 Energy/fatigue**	35	0.15 (-0.19, 0.46)	0.38
**SF-36 Emotional well-being**	35	0.27 (-0.07, 0.55)	0.12
**SF-36 General health**	35	-0.06 (-0.38, 0.28)	0.74
**SF-36 Pain**	35	0.08 (-0.26, 0.40)	0.66
**SF-36 Physical functioning**	35	-0.10 (-0.42, 0.25)	0.59
**SF-36 Role limitations due to emotional problems**	35	-0.11 (-0.43, 0.23)	0.53
**SF-36 Role limitations due to physical health**	35	0.11 (-0.23, 0.43)	0.53
**SF-36 Social functioning**	35	0.22 (-0.12, 0.52)	0.20

rho: Spearman's correlation; CI: confidence interval
